# Multi-biological activity assessment and phytochemical characterization of an aqueous extract of the *Cymbopogon citratus* grown in Palestine

**DOI:** 10.1186/s12906-024-04338-z

**Published:** 2024-01-09

**Authors:** Belal Rahhal, Mohammad Qneibi, Nidal Jaradat, Mohammed Hawash, Mohammad Qadi, Linda Issa, Sosana Bdir

**Affiliations:** 1 Department of Biomedical Sciences, Faculty of Medicine and Health Sciences, An-Najah National University, Nablus, Palestine; 2 Department of Pharmacy, Faculty of Medicine and Health Sciences, An-Najah National University, Nablus, Palestine

**Keywords:** *Cymbopogon citratus*, AMPA receptor, Plant extracts, Oxidative stress, Diabetes, Obesity, Cytotoxicity, Cancer

## Abstract

**Background:**

Plants have historically been a rich source of medicinal compounds, with many modern pharmaceuticals derived from botanical origins. In contemporary healthcare, there is a resurgence in utilizing botanical substances as recognized medicinal agents. This study delved into understanding the phytochemical makeup and the multifaceted biological activities of an aqueous extract from *Cymbopogon citratus* (*C. citratus*). The investigated activities were its effect on AMPA receptors, antioxidant capacity, anti-lipase, anti-α-amylase actions, cytotoxicity, and antimicrobial properties.

**Methods:**

The extract of *C. citratus* received a comprehensive investigation, which included the study of its phytochemical composition, assessment of its antioxidant and anti-lipase properties, evaluation of its capacity to inhibit α-amylase, analysis of its impact on cell viability, and assessment of its antimicrobial activity. The approaches are used to clarify the complex physiological and biochemical characteristics.

**Results:**

The results were compelling; receptor kinetics had a marked impact, notably on the GluA2 subunit. Regarding its medicinal potential, the extract demonstrated potent antioxidant and anti-diabetic activities with IC_50_ values of 15.13 and 101.14 µg/mL, respectively. Additionally, it displayed significant inhibitory effects on the lipase enzyme and showed cytotoxicity against the Hep3B cancer cell line, with IC_50_ values of 144.35 and 148.37 µg/mL. In contrast, its effects on the normal LX-2 cell line were minimal, indicating selectivity.

**Conclusion:**

The aqueous extract of *C. citratus* shows promising therapeutic properties. The findings advocate for further research into its compounds for potential isolation, purification, and in-depth pharmacological studies, especially in areas like nervous system disorders, diabetes, obesity, and combating oxidative stress.

**Supplementary Information:**

The online version contains supplementary material available at 10.1186/s12906-024-04338-z.

## Background

Herbal extracts have long been a vital source of therapeutic compounds, providing a significant link between nature and well-being throughout history. Herbs provide therapeutic properties used in many civilizations worldwide, serving as the foundation of traditional medical customs. Abbott offers a comprehensive analysis of the use of Hawaiian plants, ranging from their usage as food to their significance as spiritual objects [1]. Furthermore, Pradesh and UniyaJ provide insights into the localized uses of plants [2, 3], with Pradesh emphasizing their efficacy in treating certain illnesses. Pereira contributes a unique element to the diverse range of traditional cultures by exploring the use of medicinal herbs among older individuals [4]. Seniors often rely on these herbs as a safe and trustworthy therapeutic asset, highlighting their long-term significance in healthcare.

The therapeutic value of herbal extracts is smoothly integrated into contemporary medicine, as their abundant bioactive components and varied pharmacological capabilities are still being used [5, 6]. This use is especially evident in tackling current issues inside the central nervous system [7]. As we explore the complex relationship between old knowledge and current science, herbal extracts become increasingly important, connecting traditional and innovative approaches in healthcare.

Central to this exploration is the α-amino-3-hydroxy-5-methyl-4-isoxazolepropionic acid (AMPA) receptor, an ionotropic glutamate receptor in the central nervous system. AMPA receptors play a critical role in synaptic plasticity, a process underlying learning and memory. When activated by the neurotransmitter glutamate, these receptors allow cations to flow into neurons, leading to excitation and influencing neural communication [8, 9]. Their behavior and functionality are nuanced by kinetic processes such as desensitization, wherein prolonged glutamate exposure leads to receptor inactivity even in the presence of the neurotransmitter, and deactivation, where the receptor returns to its resting state post glutamate unbinding [10, 11]. Dysfunction or misregulation of AMPA receptors has been linked to various neurological conditions. Overactivity of these receptors, due to excessive glutamatergic signaling, can result in excitotoxicity—a pathological process contributing to conditions like epilepsy, stroke, and certain neurodegenerative diseases such as Alzheimer’s [12, 13]. On the other hand, their role in synaptic plasticity has made them a target of interest in mood disorders, where modulating AMPA receptor function has shown potential therapeutic benefits in depression.

In the broader context of health, shifting our gaze beyond neurological concerns, diabetes mellitus (D.M.) is a chronic metabolic disorder characterized by elevated blood sugar levels, and it has emerged as a significant global healthcare problem. With its rising prevalence, associated complications, and economic burden, D.M. is a major concern for healthcare systems worldwide [14]. The International Diabetes Federation (IDF) estimates that 463 million adults worldwide had diabetes in 2019, which will rise to 700 million by 2045. Numerous elements, such as lifestyle modifications, urbanization, and aging populations, can be blamed for this significant increase [15]. In tandem, obesity is a complex and multifaceted global health issue that necessitates a multifaceted approach. Individual choices are influenced by various environmental, economic, social, and genetic influences. To effectively combat obesity, a combination of education, policy changes, public health strategies, and a shift in societal norms regarding nutrition and physical activity is required [16].

As we delve more into the complex network of health determinants, we now focus on the widespread impact of oxidative stress, a recurring element that connects different health issues. Oxidative stress arises due to factors such as heightened metabolic rate, mitochondrial malfunction, higher cell signaling, production of oncogenes, and increased peroxisome activities. An imbalance between antioxidants and reactive oxygen species (ROS), producing peroxides and free radicals, is prevalent in several health disorders. It closely links disorders such as obesity, diabetes, cancer, and neurodegenerative diseases. In the case of obesity and diabetes, it exacerbates metabolic dysfunction; in cancer, it adds to cellular alterations; and in neurodegenerative illnesses, it interferes with neural processes. Acknowledging oxidative stress as a common cause underscores the interconnectedness of many health issues, underscoring the need for comprehensive therapies [17–20]. Effectively regulating oxidative stress by dietary and exercise modifications and appropriate medicinal therapies when required is crucial in preventing and treating many health issues. In addition, early detection and good medical intervention may reduce the risk of cancer in individuals with diabetes and obesity since these conditions are major contributors to illness and death worldwide [21]. Cancer significantly impacts individuals, families, and healthcare systems [22, 23].

Expanding our exploration to another critical facet of global health, we turn our attention to the escalating concern of antimicrobial resistance (AMR), or drug or antibiotic resistance, which refers to microorganisms’ ability to resist the effects of antimicrobial agents such as antibiotics, antivirals, antifungals, and antiparasitic drugs. When microorganisms evolve and adapt in response to the selective pressure of these medications, they become less effective or ineffective in treating infections [24].

Exploring the intersection of traditional herbal knowledge and current scientific investigation, we focus on the stems and foliage of *Cymbopogon citratus* (Lemongrass). These components have long been acknowledged for their many healing powers, deeply established in ancient medicinal practices. Lemongrass has elongated, thin green stems with a unique citrusy aroma, which is the source of its name. The plant has elongated, thin, green leaves resembling blades, enhancing its visual aspect. It releases a fragrance reminiscent of citrus, making it desirable for culinary applications, aromatherapy, and particularly traditional medicine [25, 26]. They have been recognized for their anti-inflammatory [27], antimicrobial [28], and antioxidant activities [29]. *C. Citratus* harbors a distinctive chemical composition comprising citral, geraniol, and neral as its primary bioactive components, all of which assume pivotal roles in its neuroprotective attributes [30, 31]. Furthermore, lemongrass’s antioxidant prowess, attributed to its citral content, positions it as a potential ally in mitigating oxidative stress, a common factor in neurodegenerative conditions such as Alzheimer’s and Parkinson’s diseases [32, 33]. Furthermore, the alluring capability of *C. citratus* oil also includes regulating Brain-derived neurotrophic factor (BDNF), an essential protein vital for the survival of neurons, cognitive abilities, and the creation of memories [34].

The purpose of this study is to examine the effect of *C. citratus* water extract on the activity and kinetics of AMPA receptor subunits, both homomeric (GluA1 and GluA2) and heteromeric (GluA1/2 and GluA2/3) subunits, in the setting of the nervous system. Furthermore, it intends to examine its antimicrobial, antioxidant, anti-obesity, and anti-diabetic properties, providing a nuanced perspective that blends ancient herbal knowledge with contemporary scientific research.

## Methods

### Plant collection and preparing

*C. citratus* leaves were harvested in April 2023 in Palestine’s Tubas province. Dr. Nidal Jaradat performed botanical characterization in the Pharmacognosy Laboratory at An-Najah National University and stored the specimen under the herbarium voucher number (Pharm-PCT-2780).

The leaves were washed and dried in the shade at a regulated humidity (55 ± 5 R.H.) and temperature (25 ± 2 °C). Afterward, the powdered leaf pieces were stored in airtight containers for future use.

#### The exhaustive extraction method

Approximately 25 g of powdered *C. citratus* was steeped in 500 mL of water and shaken for 72 h at room temperature at 100 rounds per minute for 7 days. The aqueous extract was filter-evaporated under specific vacuum settings using a rotavapor. A cryo-desiccator was used to lyophilize the extract. Finally, the aqueous plant extract was kept at 4 °C until further usage in the refrigerator [35].

#### Preliminary phytochemical assessment

The aqueous extracts of *C. citratus* were tested for key natural phytochemical classes using the analytical techniques described previously [36, 37].

#### Electrophysiological recordings

The study utilized HEK293t cells expressing AMPARs (flip isoform) from various subunits generously provided by S.F. Heinemann at the Salk Institute in La Jolla, CA. Transient transfections were facilitated using jetPRIME (Polyplus: New York, NY) [38]. Electrophysiological measurements were obtained via the whole-cell patch-clamp technique described in previous works [39, 40]. Integrated Patch Clamp Amplifiers with Data Acquisition System (I.P.A., Sutter Instruments, Novato, CA) were employed for data acquisition. A piezoelectric translator (Automate Scientific, Berkeley, CA) controlled a two-barrel theta glass pipette, facilitating rapid solution exchange. One barrel contained the wash solution, while the other contained the *C. citratus* solution (800 µg/ml) mixed with glutamate. The concentration of the *C. citratus* water extract was determined through incremental testing, starting at 100 µM and increasing until reaching the maximum effect without compromising cell viability (800 µg/ml). Electrode resistance was maintained between 2 and 4 µΩ. Data analysis involved fitting the current decline from 90 to 95% of the peak to the baseline current using two exponentials, allowing the calculation of time constants for deactivation (τ_w_ deact) and desensitization (τ_w_ des). The weighted tau (τ_w_) was computed as τ_w_ = (τf x af) + (τs x as), where af and as represent the amplitudes of the fast (τf) and slow (τs) exponential components, respectively. Currents of deactivation and desensitization were measured using 10 mM of glutamate for 1 ms and 500 ms, respectively, at a potential of -60 mV, pH 7.4, and room temperature (20–23 °C). Each experiment included 6 cells to calculate the mean of inhibition, desensitization, and deactivation. Control recordings were conducted with glutamate alone, ensuring that recordings before and after the application of treatment were indistinguishable from authenticating the effect of *C. citratus* solution on the cells and confirming cellular health. Data analysis was performed using Igor Pro7 (Wave Metrics, Inc). Statistical differences between experimental groups and the wild type were assessed using one-way analysis of variance (ANOVA). Significance levels were set as follows: *p < 0.05; **p < 0.01; ***p < 0.05; ns, insignificant, with *** *p* < 0.05 indicative of statistical significance.

#### Antioxidant activity

To assess the antioxidant properties of *C. citratus* extract and the positive control Trolox, a 1 mg/mL solution was initially prepared in methanol [41]. This solution served as the basis for creating a range of concentrations, including 2, 5, 7, 10, 20, 50, 80, and 100 µg/mL. Simultaneously, the DPPH**·** reagent was diluted in 0.002% w/v methanol, and these dilutions were mixed with the previously generated concentration series in a 1:1:1 ratio. As a reference, a pure methanol solution was used. Subsequently, all the solutions were incubated in a light-protected environment at room temperature for 30 min. The absorbance values were then determined using a UV-visible spectrophotometer set at a wavelength of 517 nm. Finally, the antioxidant half-maximal inhibitory concentrations (IC_50_) of *C. citratus* and Trolox were calculated using BioDataFit edition 1.02 [42].

#### Porcine pancreatic lipase inhibition assay

The anti-lipase assay was conducted by the established methodology, with minor adjustments. To summarize, a stock solution was prepared by combining 1 mg/mL of the *C. citratus* component with 10% dimethyl sulfoxide, and this stock solution was used to create various concentrations (50, 100, 200, 300, 400, and 500 µg/mL). Simultaneously, a pancreatic lipase stock solution at 1 mg/mL was mixed with a Tris-HCl buffer solution. A stock solution was created by dissolving 20.90 mg of p-nitrophenyl butyrate in 2 mL of acetonitrile. Next, 0.2 mL of the plant fraction was combined with 0.1 mL of porcine pancreatic lipase enzyme (1 mg/ml). The resulting *C. citratus* extract was further diluted to 1 mL using a Tris-HCl solution and incubated at 37 °C for 15 min. Subsequently, 0.1 mL of p-nitrophenyl butyrate was added to the *C. citratus* extract, which was then incubated at 37 °C for 30 min. The hydrolysis of p-nitrophenolate to p-nitrophenol at 405 nm was measured using a UV/visible spectrophotometer to assess pancreatic lipase activity. Furthermore, all the *C. citratus* samples and the positive control Orlistat were subjected to triplicate testing [43].

#### α–Amylase inhibitory assay

The procedure was carried out with a modified approach. In each test tube, a 200 µL sample of *C. citratus* extract and the positive control Acarbose, at concentrations of 10, 50, 70, 100, and 500 µg/mL, was combined with 200 µL of 0.02 M sodium phosphate buffer (pH 6.9) containing α-amylase solution (2 units/ml). After 10 min at 25 °C, 200 µL of 1% starch solution mixed with 0.02 M sodium phosphate buffer solution (pH 6.9) was introduced at specific intervals and allowed to react for an additional 10 min at 25 °C. The reaction was stopped by adding 200 µL of DNSA, then placing the tubes in boiling water for 5 min and cooling them to room temperature. The *C. citratus* extracts were then diluted with 5 mL of distilled water, and the absorbance at 540 nm was measured using a UV-visible spectrophotometer. A control sample was prepared following the same process, but the *C. citratus* extract was replaced with distilled water. The following equation was employed to determine the α-amylase inhibitory activity as a percentage of inhibition. Graphical analysis calculated the IC_50_, representing the amount of *C. citratus* extract components needed to inhibit α-amylase enzyme activity by 50% [44, 45].

#### Cell culture and cytotoxicity assay

Cultured cell lines, including HepG2, Hep3B, MCF-7, HeLa, CaCo-2, B16F1 tumor cells, and the normal hepatic cell line LX-2, were grown separately in RPMI-1640 media (Sigma, Norwich, UK). The media was supplemented with 1% L-glutamine (Sigma, London, UK), 1% penicillin/streptomycin antibiotics (B.I., New Delhi, India), and 10% fetal bovine serum. These cells were cultured under humid conditions with 5% CO_2_ at 37 °C. In 96-well plates, cells were seeded at a density of 2.6 × 10^4^ cells per well. After 48 h, cancer cells were exposed to *C. citratus* extract at various concentrations (50, 100, 300, 500, and 1000 µg/mL) for 24 h. Cell viability was assessed using the CellTiter 96® Aqueous One Solution Cell Proliferation (MTS.) Assay from Promega Corporation, Madison, WI, USA, following the manufacturer’s instructions. Following the treatment, 20 µL of MTS solution was added to each well containing 100 µL of medium, and the plates with *C. citratus* extract were incubated at 37 °C for 2 h. Absorbance was measured at 490 nm [46, 47].

#### Antimicrobial testing

The bacterial and fungal isolates analyzed in this study were sourced from the American Type Culture Collection (ATCC) and a clinically verified Methicillin-Resistant *Staphylococcus aureus* (MRSA) strain. The isolates consisted of two species belonging to the Gram-positive category: *Staphylococcus aureus* (ATCC 25,923) and a clinical strain of MRSA. Additionally, four species belonging to the Gram-negative category were included: *Klebsiella pneumoniae* (ATCC 13,883), *Proteus vulgaris* (ATCC 8427), *Escherichia coli* (ATCC 25,922), and *Pseudomonas aeruginosa* (ATCC 9027). In the present study, the fungal isolate utilized was *Candida albicans* (ATCC 90,028).

The antibacterial efficacy of *C. citratus* water extract was evaluated as previously described and carried out employing the broth microdilution assay [48, 49]. The *C. citratus* extract was solubilized to achieve a 100 mg/ml concentration. Subsequently, the resulting solution underwent a serial micro-dilution process, wherein it was diluted by a factor of 2, repeated 10 times, using sterile Mueller-Hinton broth (MHB). The dilution procedures were performed in a sterile setting within 96 well plates. The eleventh well consisted of MHB without any extract, a positive control for assessing microbial growth.

In contrast, well twelve was filled with MHB devoid of extracts and microbes. This particular well served as a negative control to assess the absence of microbial growth. The Wells numbers ranging from 1 to 11 were aseptically inoculated with the test microorganisms, which had been previously prepared in MHB, in order to achieve a standardized bacterial concentration. The experiment evaluating antimicrobial activity was conducted in triplicate. The plates that had been inoculated were placed in an incubator set at a temperature of 35 °C. In the case of C. albicans, a similar approach was employed, substituting RPMI medium for MHB. The incubation time was approximately 18–24 h for the plates inoculated with the test bacterial strains, whereas it extended to approximately 48 h for those inoculated with *C. albicans*. The studied extract’s lowest inhibitory concentration (MIC) was determined as the lowest concentration at which no apparent microbial growth was observed in the micro-well. The antimicrobial activity assessment involved utilizing established antimicrobial drugs, including Ampicillin and Ciprofloxacin, which are positive controls for antibacterial activity. Additionally, Fluconazole was employed as a positive control to examine antifungal activity [50].

#### Statistical analysis

The antioxidant, anti-lipase, anti-α-amylase, and cytotoxic activities of the *C. citratus* extract and positive controls were assessed, and the results are presented as the mean ± standard deviation (S.D.). Significance was determined when the p-value reached 0.05.

## Results

### Phytochemical screening

As indicated in Table 1, preliminary phytochemical screening revealed the presence of tannins, phenols, saponin, polysaccharides, and flavonoids as important phytoconstituents in the aqueous extract of *C. citratus*.

**Table 1:** Phytochemical screening of the aqueous extract of *C. citratus*

Major classes	Results
Alkaloids	-ve
Carbohydrates	+ve
Sugar	-ve
Glycosides	+ve
Saponin	+ve
Phenols	+ve
Tannins	+ve
Flavonoids	+ve
Protein	-ve

### Effect of *C. citratus* water extract on AMPA receptor subunits activity and kinetics

**Fig. 1:**
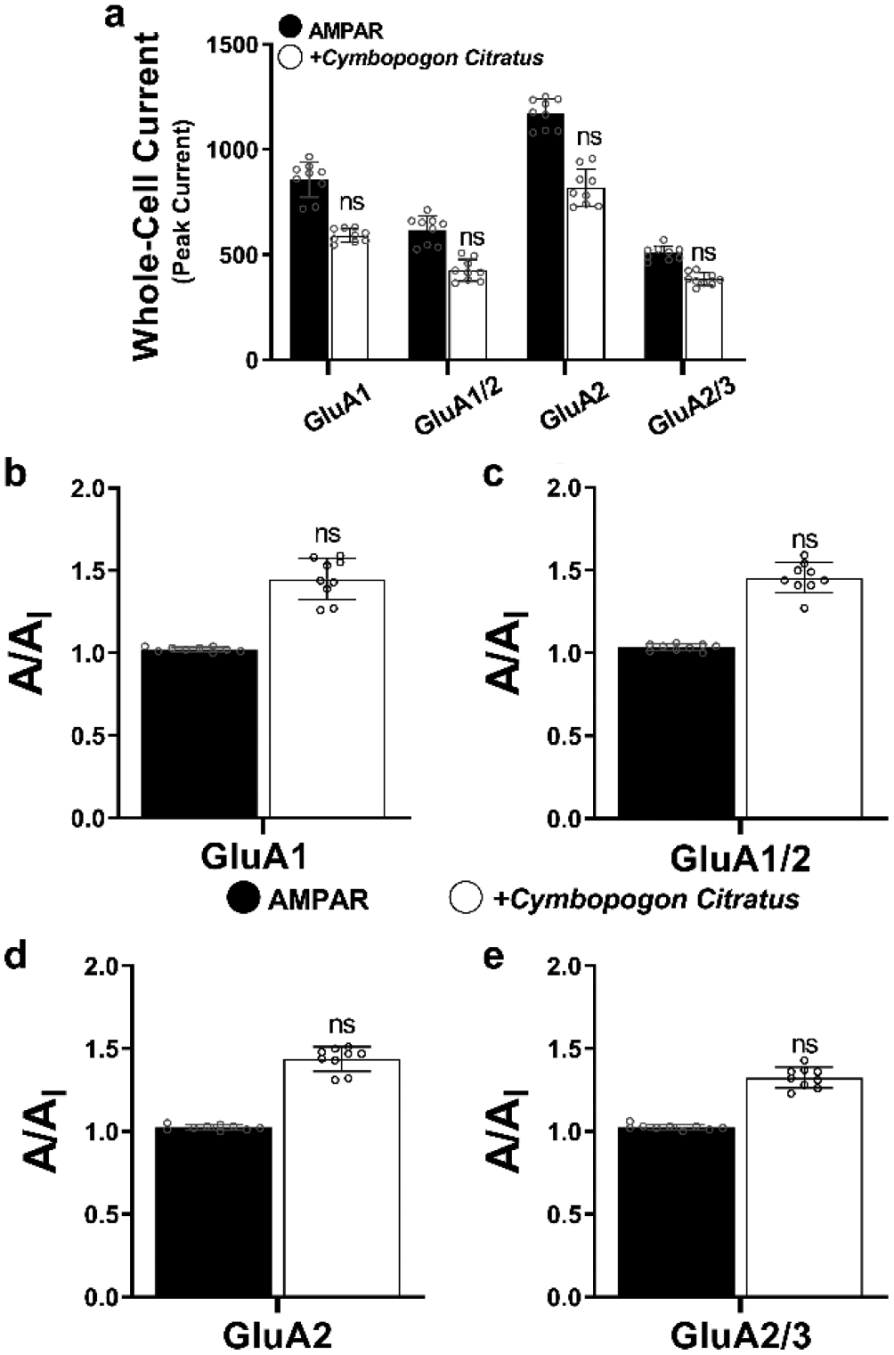
Whole-cell current responses of AMPA receptor subunits following exposure to *C. citratus* water extract. Panel (**a**) illustrates the whole-cell current responses (in pA) of AMPA receptor subunits expressed in HEK293t cells, both in the presence of glutamate alone (black) and after the administration of 800 µg/ml of *C. citratus* extract (white). To provide a comprehensive view of the impact, Figures (**b, c, d**), and (**e**) present the A/A_I_ ratio, where ‘A’ represents the current response to glutamate alone, and ‘A._I_.’ signifies the current response following the introduction of the *C. citratus* water extract. Notably, all whole-cell current measurements were obtained at -60 mV, under pH 7.4, and at a temperature of 22 °C. Statistical comparisons were conducted using one-way analysis of variance (ANOVA), with ‘ns’ indicating non-significance. Each experimental dataset is based on evaluating 6 cells, and all data are presented as means ± SEM. The dots plotted above each column depict the current generated by each cell of the 6 cells examined following the extract’s application

Our investigation commenced by examining the impact of *C. citratus* water extract on the activity of AMPA receptor subunits, encompassing both homomeric and heteromeric configurations, specifically GluA1, GluA2, GluA1/2, and GluA2/3. Intriguingly, the results revealed that the application of *C. citratus* water extract at a concentration of 800 µg/ml led to a subtle reduction in whole-cell current for the tested subunits, although this effect did not reach the threshold of Inhibition (Fig. 1, a). To provide a deeper perspective, we introduced the concept of the A/A_I_ ratio, comparing the currents induced by glutamate (A) with those following *C. citratus* extract administration (A._I_.) (Fig. 1, b, c, d, and e).

**Fig. 2:**
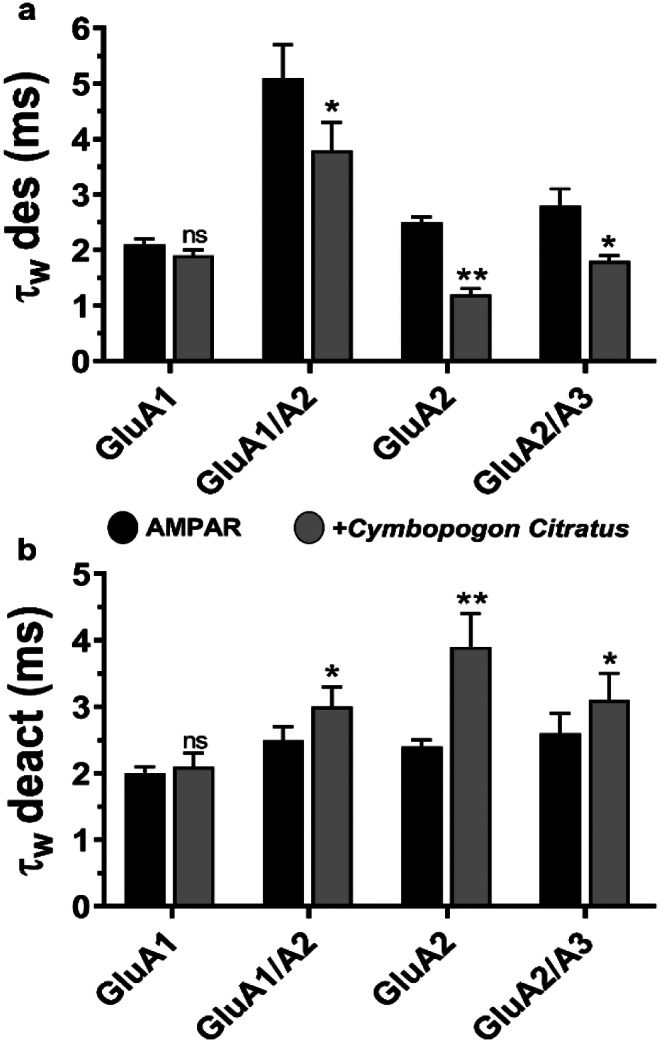
*C. citratus* effect on AMPA receptor desensitization and deactivation rates. (**a**) Shows τ_w_ des in the presence of glutamate alone (black) and *C. citratus* (gray). The traces of the four tested subunits were recorded using the 500 ms protocol to detect the desensitization rate. (**b**) shows τ_w_ deact in the presence of glutamate alone (black) and *C. citratus* (gray). The traces of the four tested subunits using the 1 ms protocol to detect the deactivation rate. The currents were measured at -60 mV, pH 7.4, and 22 °C. For comparison, one-way analysis of variance (ANOVA) was used: *p < 0.05; **p < 0.01, ns, not significant. In each experiment, 6 cells were evaluated, and all data are provided as means ± SEM

As our investigation delved further into the impact of *C. citratus* extract on AMPA receptor subunit activity, we employed the whole-cell patch clamp technique to record deactivation rates (τ_w_ deact) and desensitization rates (τ_w_ des). Notably, the *C. citratus* solution moderately influenced the kinetics, with the most prominent effect observed in the GluA2 subunit. GluA2 exhibited a 1.6-fold increase in deactivation rate (Fig. 2, b), coupled with a 2-fold decrease in desensitization rate (Fig. 2, a). Similarly, when examining heteromeric subunits, we found a 1.5-fold reduction in desensitization rate and a 1.2-fold increase in deactivation rate (Fig. 2). These intriguing findings shed light on the nuanced impact of *C. citratus* extract on AMPA receptor subunit kinetics.

### Antioxidant, anti-lipase, anti-α-amylase, and cytotoxicity

**Fig. 3:**
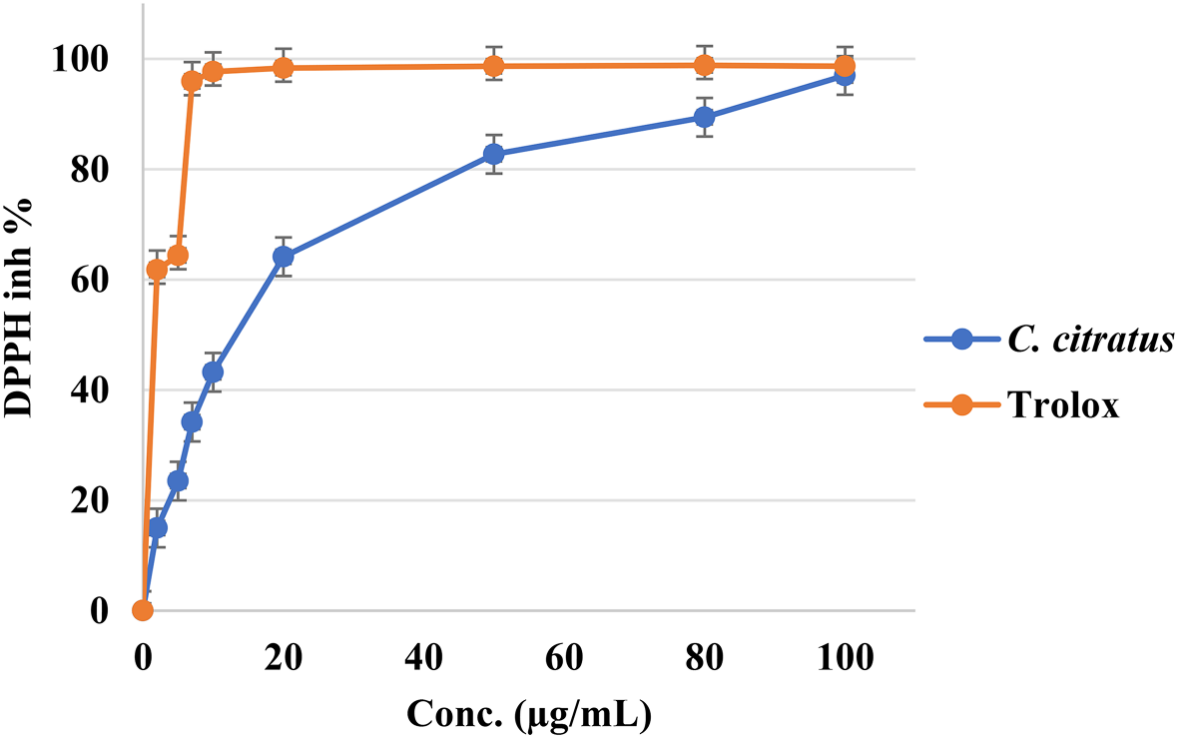
DPPH free radicals scavenging property of the *C. citratus* extract and Trolox

The antioxidant activities of the *C. citratus* extract were evaluated using the DPPH**·** assay, and the results are displayed in Fig. 3. The *C. citratus* extract exhibited an IC_50_ value of 15.13 µg/mL, compared to a positive control (Trolox) with an IC_50_ of 1.54 µg/mL, as indicated in Table 2.

**Table 2:** The IC_50_ Values (µg/mL) on each biological target of *C. citratus* aqueous extract in comparison with the positive control

	IC_50_ (µg/mL)	
Biological Targets	*C. citratus*	Control
α-Amylase	101.14 ± 2.21	7.66 ± 1.78^a^
Lipase	144.35 ± 2.15	6.56 ± 1.55^b^
DPPH	15.13 ± 1.19	1.54 ± 1.42^c^
HepG2	381.30 ± 1.29	4.23 ± 1.78^d^
CaCo-2	430.49 ± 1.76	3.71 ± 0.84^d^
Hep3B	148.37 ± 2.45	1.21 ± 1.0^d^
MCF-7	468.03 ± 2.29	1.28 ± 0.45^d^
HeLa	555.18 ± 2.15	2.09 ± 1.01^d^
B16F1	827.62 ± 2.42	12.91 ± 1.36^d^
LX-2	> 1000	0.73 ± 0.33^d^

^a^Acarbose, ^b^Orlistat, ^c^Trolox, and ^d^5-Fu while the p-value was < 0.05

The percentage of inhibition was measured for the *C. citratus* extract at different concentrations, and the results are illustrated in Fig. 3. The *C. citratus* extract showed a significantly high percentage of inhibition; it was too close to the IC_50_ of the control (Trolox).

The MTS assay was employed to assess the used to determine the antiproliferative or cytotoxic effects of the *C. citratus* extract. The results revealed significant antiproliferative properties of the *C. citratus* extract, particularly against liver cancer HepG2, with an IC_50_ value of 381.30 µg/mL, in contrast to its effects on normal cells with an IC_50_ value exceeding 1000 µg/mL. The IC_50_ values for other cancer cell lines, compared to the positive control anti-cancer drug 5-Fu, are presented in Table 2.

**Fig. 4:**
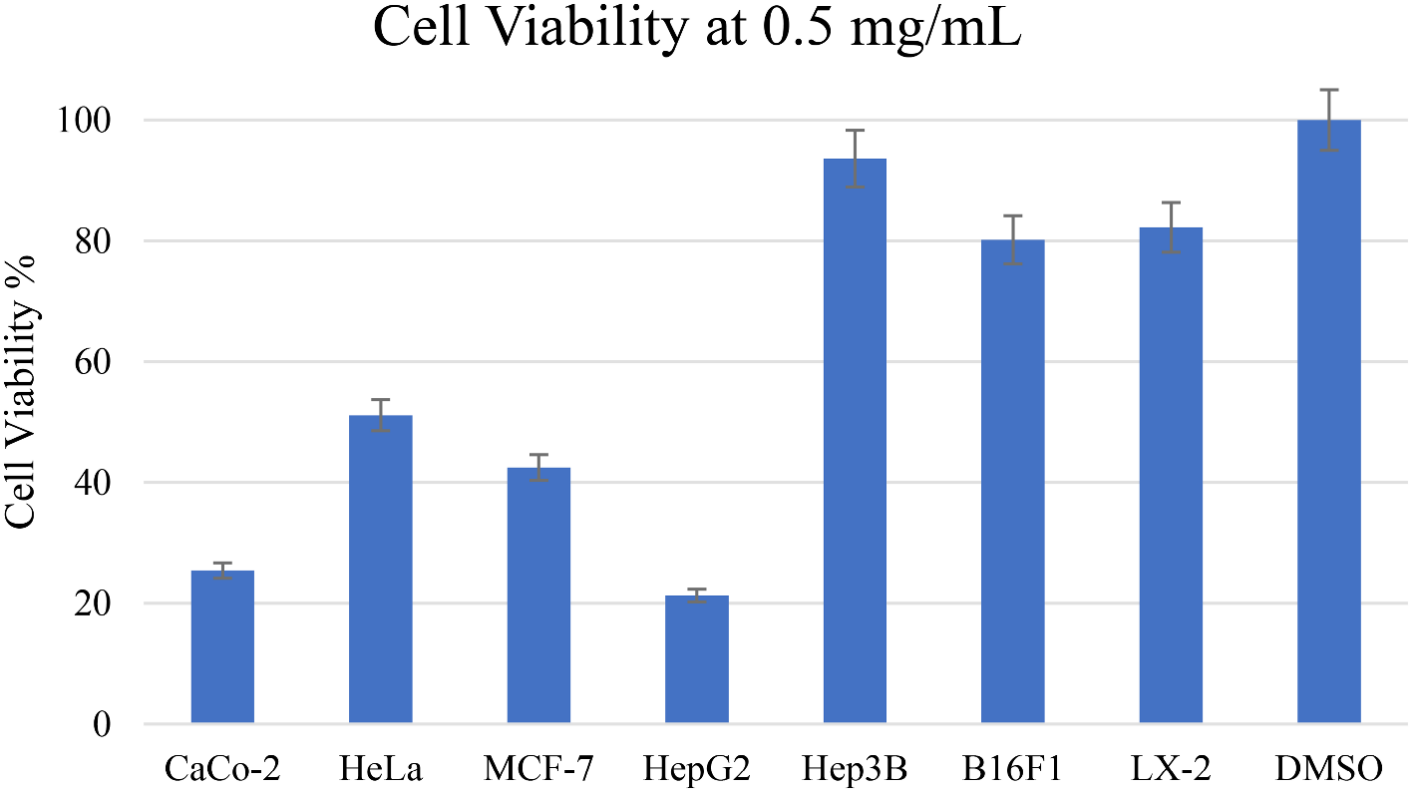
Cytotoxic activity of *C. citratus* extract and negative control DMSO on cancer and normal cell lines

Additionally, Fig. 4 illustrates the cell viability of the *C. citratus* extract at a concentration of 500 µg/mL. Notably, at this concentration, the *C. citratus* extract displayed lower cell viability for cancer cell lines HepG2 and CaCo-2, with values of 21.29% and 25.34%, respectively, as opposed to the higher viability of the normal cell line LX-2, which exhibited a value of 82.24%.

**Fig. 5:**
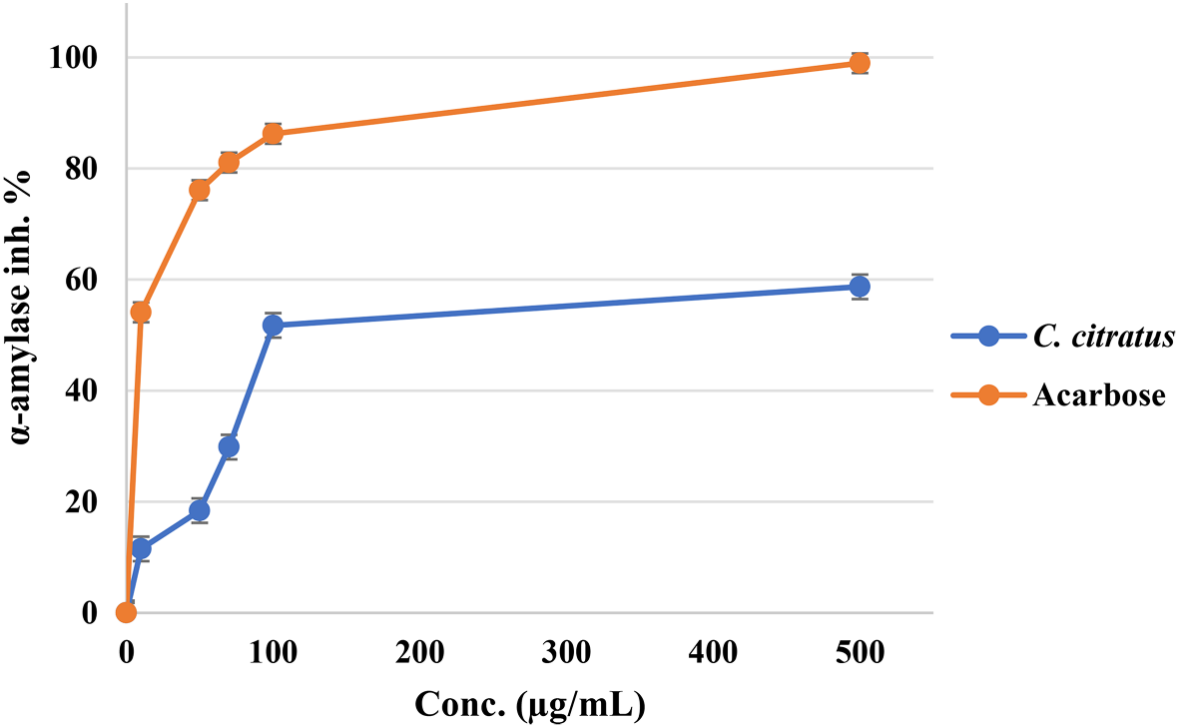
Anti-α-amylase effect of *C. citratus* extract and Acarbose

**Fig. 6:**
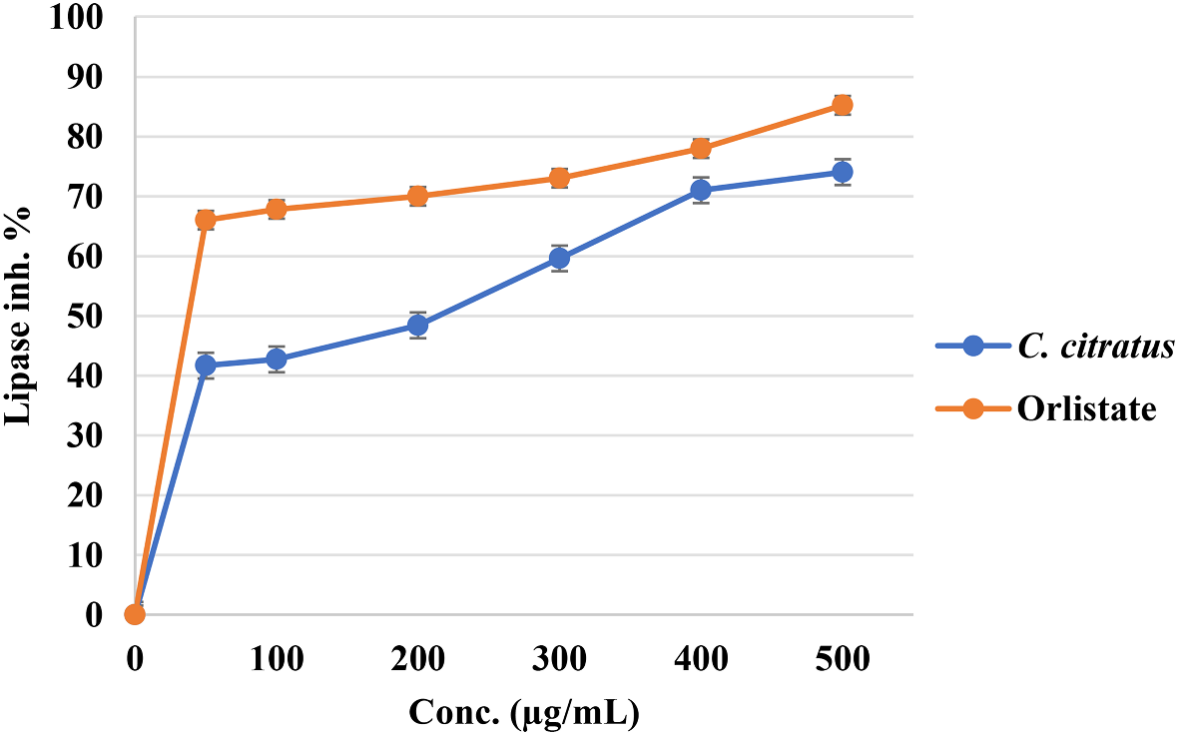
Antilipase effect of *C. citratus* extract and Orlistat

The anti-diabetic and anti-obesity potential of the evaluated *C. citratus* extract was evaluated by examining their effects on α-amylase and lipase enzymes. The results of IC_50_ values were presented in Table 2; the *C. citratus* extract exhibited potent anti-amylase activity with an IC_50_ value of 101.14 µg/mL; in contrast, the control drug Acarbose displayed an IC_50_ value of 7.66 µg/mL, indicating that *C. citratus* extract has potent anti-amylase effects in comparison with the other plants extracts. However, this extract significantly affected lipase activity, which exhibited IC_50_ values of 144.35 µg/mL, whereas the control drug Orlistat had an IC_50_ value of 6.56 µg/mL, as shown in Table 2. The inhibitory activity percentages of the various concentrations conducted against these two enzymes were presented in Figs. 5 and 6 accordingly. These findings suggest that the evaluated extract could be used as an effective natural treatment for diabetes and obesity due to their significant anti-amylase activity with caution because of their potent activities on various cell lines.

### Antimicrobial testing

In our investigation, *C. citratus* water extract demonstrated no potential antibacterial activity against multiple gram-negative and gram-positive bacterial species and no antifungal activity against the fungal species employed in this study, *C. albicans*, as indicated in Table 3.

**Table 3:** Antimicrobial activity minimal inhibitory concentration (MIC) values (mg/ml) of *C. citratus* aqueous extract and antibiotics

*C. citratus*	Bacteria						Fungus
	**Gram-positives**		**Gram-negative**				**Yeast**
**ATCC Number**	**Clinical strain**	**ATCC 25,923**	**ATCC 25,922**	**ATCC 13,883**	**ATCC 8427**	**ATCC 9027**	**ATCC 90,028**
Microbe	MRSA	*S. aureus*	*E. coli*	*Klebsiella pneumoniae*	*Proteus vulgaris*	*Pseudomonas aeruginosa*	*Candida albicans*
M.I.C. (mg/ml)	Resistant	Resistant	Resistant	Resistant	Resistant	Resistant	Resistant

This highlights the importance of recognizing *C. citratus’s* water extract limits as an antibacterial agent. Our findings show that the water-soluble extract did not display potent antibacterial activity when tested using the micro broth dilution technique. In contrast to the water-soluble extract of *C. citratus*, several studies have investigated the antibacterial activity of *C. citratus* extracts prepared using other solvents [28]. Notably, due to high quantities of components such as citral and limonene, *C. citratus* essential oil obtained by steam distillation has demonstrated substantial antibacterial activity. This essential oil possesses antibacterial and antifungal characteristics that are effective against various microorganisms [28, 51, 52].

Furthermore, ethanolic extracts of *C. citratus* have shown significant antibacterial activity owing to their capacity to collect a larger range of bioactive chemicals [53]. The findings show that the solvent and extraction method used substantially impact the antibacterial activity of *C. citratus* extracts. While the water-soluble extract may not have as strong an effect, other *C. citratus* extracts have shown promising outcomes, underscoring the necessity of choosing the right extraction process to use its antibacterial properties fully.

## Discussion

We investigated the water-based extract of *C. citratus*, revealing its wide-ranging effects on several biological functions. We focused our examination on phytochemicals, which are plant secondary metabolites known for their medicinal potential. In a recent review conducted by Kiani et al. in 2022, lemongrass’s chemical composition and medicinal capabilities were thoroughly explained. The review emphasized the well-established qualities of lemongrass, including its ability to act as an antioxidant, antimicrobial, anti-inflammatory, anti-hypertensive, anti-diabetic, anti-mutagenic, and anxiolytic agent. Additionally, lemongrass was found to possess hypoglycemic and hypolipidemic activities. Our work supports these previous results, confirming the strong consistency in many studies [54]. These findings have significant implications for applications in neurobiology, metabolic disorders, and oxidative stress management.

The study began by examining the influence of *C. citratus* water extract on AMPA receptor subunits. At a concentration of 800 µg/ml, the extract induced a subtle reduction in whole-cell current for the tested subunits, although it did not reach the inhibition threshold. This subtle effect hints at the potential modulation of neurotransmission, relevant in conditions like epilepsy where excessive excitatory neurotransmission can be problematic. Furthermore, introducing the A/A_I_ ratio provided a deeper perspective into how *C. citratus* extract affects synaptic activity and excitatory neurotransmission. It highlights the nuanced impact of the extract on neural signaling and its potential role in maintaining balanced synaptic activity.

The research demonstrated that the extract from *C. citratus* significantly impacted the behavior of AMPA receptor subunits, particularly GluA2. Upon exposure to the extract, the GluA2 subunit exhibited a notable augmentation in its deactivation rate and a halving of its desensitization rate. This implies that AMPA receptors may return quicker to their baseline state after glutamate binding, indicating an improved deactivation process. Additionally, they can stay receptive to stimuli without becoming excessively active, as seen by decreased desensitization. The control of kinetic properties, particularly in the GluA2 subunit, is essential since it governs how much calcium may pass through AMPA receptors. Modifications in its conduct may impact the intracellular fluctuations of calcium, which are crucial for a range of neurophysiological functions, including synaptic plasticity.

The extract of *C. citratus* can influence the rate of the receptor’s activity, which may contribute to maintaining a stable synaptic activity. This ability may have the potential to avoid excitotoxic damage, a harmful process linked to neurodegenerative disorders such as Alzheimer’s disease. Moreover, the rapid inactivation of receptors corresponds to *C. citratus’s* known impact on BDNF production, which plays a vital role in brain cells’ survival, cognition, and memory. The findings highlight the potential of *C. citratus* in modulating neurotransmission and affecting cellular signaling pathways that depend on calcium dynamics.

Acknowledging the possible synergy between the main bioactive constituents of *C. citratus* extract - citral, geraniol, and neral is crucial. Although our research primarily focused on the overall impact of *C. citratus* extract, comprehending these constituents’ individual and combined contributions might provide insight into underlying processes. Due to its strong antioxidant properties, citral may defend against oxidative stress, a frequent contributor to neurodegenerative disorders [55]. Geraniol and neral may be crucial in regulating the speed at which AMPA receptors function. The results indicate that the *C. citratus* extract has the potential to be used as a therapy for these illnesses, particularly due to its proven anticonvulsant properties in prior in-vivo research [56]. This finding establishes the extract as a promising option for future investigation in neurotherapeutic research.

In addition to investigating brain signaling, the research also examined the antioxidant capabilities of the *C. citratus* extract using the DPPH test. Although the IC_50_ value exceeded that of the positive control, Trolox, the extract demonstrated a greater percentage of inhibition than the control, highlighting its potential for effectively treating oxidative stress. Moreover, the extract had notable anti-cancer characteristics, specifically targeting liver cancer cells (HepG2), and it indicated a preference for cancer cells, which justifies the need for further exploration of the underlying processes. *C. citratus* has antioxidant capabilities that may protect against various illnesses caused by oxidative stress, such as neurological disorders. The possibility of these extracts to reduce oxidative damage at the cellular level offers a promising avenue for future therapeutic investigation. The results of our investigation are consistent with other research that has shown the antioxidant and anti-inflammatory characteristics of *C. citratus*. For instance, research conducted by Deeksha Salaria et al. emphasized the extract’s comparable antioxidant capacities, indicating its potential in treating illnesses associated with oxidative stress [57]. Nevertheless, our investigation broadens this understanding by examining the precise interactions with AMPA receptor subunits, a topic that has yet to be widely addressed in previous studies. This fresh perspective substantially contributes to the current knowledge base, proposing new avenues for therapeutic study.

The research investigated the inhibitory effects of the extract on lipase and α-amylase enzymes, which are relevant to metabolic diseases. The substance had strong inhibitory effects on amylase, indicating a possible use in the control of diabetes. Furthermore, previous studies have shown the inhibitory impact of *C. citratus* on lipase activity [58]. However, our study provides a more comprehensive elucidation of these actions at a molecular level, enhancing our knowledge of their potential in managing metabolic disorders.

Regarding the antibacterial and antifungal activities, the water-soluble *C. citratus* extract did not exhibit potent effects against various bacterial species or the fungal species *C. albicans*. This result underscores the importance of considering the choice of solvent and extraction method when assessing the antimicrobial properties of *C. citratus* extracts. It highlights that other extraction methods and solvents have shown promising results, suggesting that *C. citratus’*s potential in this context highly depends on the chosen approach. The antibacterial activity shown in our investigation aligns closely with findings from previous studies. For instance, Danlami et al. (2011) found that the ethanolic extract of *C. citratus* exhibited notably potent antimicrobial effects against *S. aureus* and *Bacillus cereus* [59].

Although our work provides insight into the favorable impacts of *C. citratus* extract, it is important to acknowledge that these findings are based on experiments conducted in a controlled laboratory environment. Therefore, more investigations involving live organisms and human subjects are necessary to validate these results in real-life conditions and assess the safety of this extract. Future research should specifically prioritize investigating the effects of the extract on neurological illnesses, examining its therapeutic capacity, and determining the most effective dosage via clinical trials. Furthermore, the notable antioxidant and anti-cancer characteristics discovered, specifically with liver cancer cells, justify further research to comprehend the underlying processes and effectiveness in cancer treatment. Comprehensive studies are needed to evaluate the long-term effects and efficacy of the extract in controlling metabolic illnesses such as diabetes. Furthermore, the variation in antibacterial characteristics depending on the extraction procedures implies the need to investigate alternative strategies to optimize effectiveness. Finally, an important topic for future investigation is the optimization of dose and the comprehension of possible interactions with current drugs, which is crucial for incorporating this extract into treatment plans.

## Conclusions

In conclusion, this study has provided a comprehensive and all-encompassing view of the many attributes of *C. citratus* extract. The potential to regulate brain signaling and its impact on synaptic plasticity and calcium dynamics is evident. It can gently adjust AMPA receptor subunits and change receptor kinetics, particularly in the GluA2 subunit. The extract’s antioxidant properties show promise in combating oxidative stress, while its distinct anti-cancer mechanisms provide prospects for further exploration in developing novel cancer therapies. The article highlights the potential roles of anti-amylase treatments in managing diabetes and underscores the need for more research on their effects on lipase activity. The absence of antibacterial and antifungal activity in the water-soluble extract of *C. citratus* emphasizes the need to choose a suitable solvent and extraction method since other extracts have shown promising results. These comprehensive findings improve our understanding of the potential applications of *C. citratus* extract in the brain, cancer research, and metabolic illnesses. An in-depth examination of the interactions between functional groups in the extract is required to reveal the underlying mechanisms and their potential uses in various medical contexts. The extract of *C. citratus* is versatile, encouraging opportunities for more scientific exploration and potential therapeutic applications.

### Electronic supplementary material

Below is the link to the electronic supplementary material.

Supplementary Material 1: A supplementary document showcasing comprehensive whole-cell recording data for HEK293t cells

## Data Availability

Data collected or analyzed in this investigation are included in this manuscript and its supplemental material file.
