# Supplementary material for: Multi-biological activity assessment and phytochemical characterization of an aqueous extract of the Cymbopogon citratus grown in Palestine
Source: BMC Complement Med Ther. 2024 Jan 9;24:27. doi: 10.1186/s12906-024-04338-z (PMC10775582; doi:10.1186/s12906-024-04338-z)
Supplement: Supplementary file 1 — Supplementary Material 1: A supplementary document showcasing comprehensive whole-cell recording data for HEK293t cells [file 12906_2024_4338_MOESM1_ESM.docx]

**Supplementary material**

**Multi-Biological Activity Assessment and Phytochemical Characterization of an Aqueous Extract of the *Cymbopogon citratus* Grown in Palestine**

**Belal Rahhal^a,*,†^, Mohammad Qneibi^a,*,†^, Nidal Jaradat^b^, Mohammed Hawash^b^, Mohammad Qadi^a^, Linda Issa^b^ and Sosana Bdir^a^**

^a^ Department of Biomedical Sciences, Faculty of Medicine and Health Sciences, An-Najah National University, Nablus, Palestine.

^b^ Department of Pharmacy, Faculty of Medicine and Health Sciences, An-Najah National University, Nablus, Palestine.

**^†^**Authors contributed equally to this work.

^*^Correspondence: [belalrahhal@najah.edu](mailto:belalrahhal@najah.edu); [mqneibi@najah.edu](mailto:mqneibi@najah.edu)

# **Table of Contents**

**Table S1. Whole-Cell Recordings…………………………………………………...…………..3**

**Table S2. The Raw data of DPPH results regarding the C. citratus and positive control..…4**

**Table S3. The Raw data of Lipase inhibitory results regarding the C. citratus and positive control ………………………………………………..……………………………………….…5**

**Table S4. The Raw data of Amylase inhibitory results regarding the C. citratus and positive control ………………………………………………………………………………….5**

**Table S5. The Raw data of cancer and normal cell liens absorbance after treated with different concentrations of C. citratus …………………………………………………………6**

**Table S1. Whole-Cell Recordings**

The presented data represents the mean value and the standard error of the mean (SEM). This study's sample size is n = 6, which refers to the number of patch cells in the whole-cell arrangement. The statistical significance of the data was assessed using a one-way analysis of variance (ANOVA) test. The significance levels were established: * p < 0.05, ** p < 0.01, and ns indicating non-significance.

| **Receptor Name/Compounds abbreviation** | **GluA1 (Glutamate Alone)** | ***Cymbopogon Citratus*** | **Applying Glutamate Alone After Cymbopogon Citratus** | **n** | **A/A_I_** |
| --- | --- | --- | --- | --- | --- |
| **Amplitude** | 857±84 | 592±31^ns^ | 837±83 | 6 | 1.45±0.1 |
| **(pA)** |  |  |  |  |  |
| **t deact** | 2.0±0.1 | 2.1±0.2^ns^ | N/R | 6 | N/R |
| **(ms)** |  |  |  |  |  |
| **t des** | 2.1±0.1 | 1.9±0.1^ns^ | N/R | 6 | N/R |
| **(ms)** |  |  |  |  |  |
| **Receptor Name/Compounds abbreviation** | **GluA1/2 (Glutamate Alone)** | ***Cymbopogon Citratus*** | **Applying Glutamate Alone After Cymbopogon Citratus** | **n** | **A/A_I_** |
| **Amplitude** | 618±66 | 426±52^ns^ | 599±68 | 6 | 1.46±0.1 |
| **(pA)** |  |  |  |  |  |
| **t deact** | 2.5±0.2 | 3.0±0.3^*^ | N/R | 6 | N/R |
| **(ms)** |  |  |  |  |  |
| **t des** | 5.1±0.6 | 3.8±0.5^*^ | N/R | 6 | N/R |
| **(ms)** |  |  |  |  |  |
| **Receptor Name/Compounds abbreviation** | **GluA2 (Glutamate Alone)** | ***Cymbopogon Citratus*** | **Applying Glutamate Alone After Cymbopogon Citratus** | **n** | **A/A_I_** |
| **Amplitude** | 1172±70 | 820±88^ns^ | 1145±69 | 6 | 1.44±0.1 |
| **(pA)** |  |  |  |  |  |
| **t deact** | 2.4±0.1 | 3.9±0.5^**^ | N/R | 6 | N/R |
| **(ms)** |  |  |  |  |  |
| **t des** | 2.5±0.1 | 1.2±0.1^**^ | N/R | 6 | N/R |
| **(ms)** |  |  |  |  |  |
| **Receptor Name/Compounds abbreviation** | **GluA2/3 (Glutamate Alone)** | ***Cymbopogon Citratus*** | **Applying Glutamate Alone After Cymbopogon Citratus** | **n** | **A/A_I_** |
| **Amplitude** | 508±33 | 384±30^ns^ | 496±31 | 6 | 1.32±0.1 |
| **(pA)** |  |  |  |  |  |
| **t deact** | 2.6±0.3 | 3.1±0.4^*^ | N/R | 6 | N/R |
| **(ms)** |  |  |  |  |  |
| **t des** | 2.8±0.3 | 1.8±0.1^*^ | N/R | 6 | N/R |
| **(ms)** |  |  |  |  |  |

**Table S2. The Raw data of DPPH results regarding the C. citratus and positive control**

|  | ***Absorbance*** | | | | | |
| --- | --- | --- | --- | --- | --- | --- |
|  | ***C. citratus*** | | | **Trolox** | | |
| con(µg/ML) | abs1 | abs2 | abs3 | abs1 | abs2 | abs3 |
| 2 | 0.226 | 0.226 | 0.228 | 0.079 | 0.077 | 0.078 |
| 5 | 0.205 | 0.204 | 0.206 | 0.072 | 0.073 | 0.073 |
| 7 | 0.176 | 0.176 | 0.177 | 0.011 | 0.012 | 0.012 |
| 10 | 0.152 | 0.152 | 0.152 | 0.005 | 0.007 | 0.006 |
| 20 | 0.095 | 0.096 | 0.097 | 0.003 | 0.003 | 0.004 |
| 50 | 0.046 | 0.046 | 0.047 | 0.002 | 0.003 | 0.003 |
| 80 | 0.028 | 0.028 | 0.029 | 0.002 | 0.002 | 0.003 |
| 100 | 0.009 | 0.008 | 0.007 | 0.001 | 0.002 | 0.001 |
|  | **Inhibition %** | | | | | |
|  | ***C. citratus*** | | | **Trolox** | | |
| con(µg/mL) | inh%1 | inh%2 | inh%3 | inh%1 | inh%2 | inh%3 |
| 2 | 15% | 15% | 14.92% | 61.27% | 62.25% | 61.76% |
| 5 | 23.50% | 23.88% | 23.13% | 64.70% | 64.21% | 64.21% |
| 7 | 34.30% | 34.30% | 33.95% | 94.60% | 96.56% | 96.56% |
| 10 | 43.20% | 43.20% | 43.20% | 97.54% | 98.52% | 97% |
| 20 | 64.50% | 64.17% | 63.80% | 98.52% | 98.52% | 98% |
| 50 | 82.80% | 82.83% | 82.46% | 99% | 98.52% | 98.52% |
| 80 | 89.55% | 89.55% | 89.17% | 99% | 99% | 98.52% |
| 100 | 96.60% | 97% | 97.38% | 98.52% | 99% | 98.52% |

**Table S3. The Raw data of Lipase inhibitory results regarding the C. citratus and positive control**

|  | ***Absorbance*** | | | ***Absorbance*** | | | |
| --- | --- | --- | --- | --- | --- | --- | --- |
|  | ***C. citratus*** | | |  | Orlistat | | |
| Con (µg/mL) | abs1 | abs2 | abs3 | Con (µg/mL) | abs1 | abs2 | abs3 |
| 50 | 0.207 | 0.206 | 0.205 | 50 | 0.129714 | 0.108758 | 0.11928 |
| 100 | 0.2 | 0.202 | 0.201 | 100 | 0.133318 | 0.105236 | 0.100357 |
| 200 | 0.181 | 0.18 | 0.182 | 200 | 0.10169 | 0.098225 | 0.115718 |
| 300 | 0.143 | 0.141 | 0.141 | 300 | 0.108253 | 0.090739 | 0.084738 |
| 400 | 0.102 | 0.1 | 0.101 | 400 | 0.086874 | 0.075285 | 0.069396 |
| 500 | 0.092 | 0.091 | 0.09 | 500 | 0.062909 | 0.041115 | 0.051235 |
|  | **Inhibition %** | | |  | **Inhibition %** | | |
| Con (µg/mL) | inh%1 | inh%2 | inh%3 | Con (µg/mL) | inh%1 | inh%2 | inh%3 |
| 50 | 41.02 | 41.31 | 42.73 | 50 | 63.0444 | 69.0147 | 66.01713 |
| 100 | 43.01 | 42.45 | 42.73 | 100 | 62.0178 | 70.01821 | 71.40833 |
| 200 | 48.43 | 48.71 | 48.14 | 200 | 71.02854 | 72.0158 | 67.03203 |
| 300 | 59.25 | 59.82 | 59.82 | 300 | 69.15874 | 74.1485 | 75.85806 |
| 400 | 70.94 | 70.94 | 71.22 | 400 | 75.2495 | 78.5514 | 80.2291 |
| 500 | 73.78 | 74.07 | 74.35 | 500 | 82.0772 | 88.2864 | 85.4032 |

**Table S4. The Raw data of Amylase inhibitory results regarding the C. citratus and positive control**

|  | ***Absorbance*** | | |  | ***Absorbance*** | | |
| --- | --- | --- | --- | --- | --- | --- | --- |
|  | ***C. citratus*** | | | Orlistat | | | |
| Con (µg/mL) | abs1 | abs2 | abs3 | Con (µg/mL) | abs1 | abs2 | abs3 |
| 10 | 0.381 | 0.381 | 0.382 | 10 | 0.208783 | 0.176618 | 0.207957 |
| 50 | 0.352 | 0.351 | 0.352 | 50 | 0.090416 | 0.103372 | 0.115239 |
| 70 | 0.301 | 0.302 | 0.304 | 70 | 0.081894 | 0.081828 | 0.081302 |
| 100 | 0.207 | 0.209 | 0.208 | 100 | 0.059272 | 0.055938 | 0.062694 |
| 500 | 0.178 | 0.177 | 0.179 | 500 | 0.000425 | 0.010874 | 0.002407 |
|  | **Inhibition %** | | |  | **Inhibition %** | | |
| Con (µg/mL) | inh%1 | inh%2 | inh%3 | Con (µg/mL) | inh%1 | inh%2 | inh%3 |
| 10 | 11.6 | 11.6 | 11.36 | 10 | 51.5584 | 59.0214 | 51.7502 |
| 50 | 18.32 | 18.56 | 18.32 | 50 | 79.0217 | 76.0158 | 73.2625 |
| 70 | 30.16 | 29.93 | 29.46 | 70 | 80.9991 | 81.0144 | 81.1365 |
| 100 | 51.97 | 51.5 | 51.74 | 100 | 86.2478 | 87.0214 | 85.4538 |
| 500 | 58.7 | 58.93 | 58.46 | 500 | 99.9014 | 97.4771 | 99.4415 |

**Table S5. The Raw data of cancer and normal cell liens absorbance after treated with different concentrations of C. citratus**

| Date:24/08/2023 |  |  |  |  |  |  |  |
| --- | --- | --- | --- | --- | --- | --- | --- |
| type of cell: CaCO-2 |  |  |  |  |  |  |  |
|  | **negative control** | **1000µg/ml** | **500µg/ml** | **300µg/ml** | **100µg/ml** | **50µg/ml** | **blank** |
| **abs1** | **1.459** | **0.300** | **0.438** | **1.461** | **1.504** | **1.578** | **0.105** |
| **abs2** | **1.478** | **0.302** | **0.465** | **1.453** | **1.528** | **1.565** | **0.104** |
|  |  |  |  |  |  |  |  |
| Date:24/08/2023 |  |  |  |  |  |  |  |
| type of cell:Hela |  |  |  |  |  |  |  |
|  | **negative control** | **1000µg/ml** | **500µg/ml** | **300µg/ml** | **100µg/ml** | **50µg/ml** | **blank** |
| **abs1** | **1.337** | **0.183** | **0.718** | **1.040** | **1.045** | **1.147** | **0.105** |
| **abs2** | **1.326** | **0.189** | **0.746** | **1.066** | **1.067** | **1.160** | **0.104** |
|  |  |  |  |  |  |  |  |
| Date:24/08/2023 |  |  |  |  |  |  |  |
| type of cell:MCF-7 |  |  |  |  |  |  |  |
|  | **negative control** | **1000µg/ml** | **500µg/ml** | **300µg/ml** | **100µg/ml** | **50µg/ml** | **blank** |
| **abs1** | **1.078** | **0.188** | **0.526** | **1.081** | **1.069** | **1.087** | **0.105** |
| **abs2** | **1.097** | **0.185** | **0.518** | **1.038** | **1.094** | **1.098** | **0.104** |
|  |  |  |  |  |  |  |  |
| Date:24/08/2023 |  |  |  |  |  |  |  |
| type of cell:Hep-G2 |  |  |  |  |  |  |  |
|  | **negative control** | **1000µg/ml** | **500µg/ml** | **300µg/ml** | **100µg/ml** | **50µg/ml** | **blank** |
| **abs1** | **1.066** | **0.184** | **0.313** | **0.906** | **1.033** | **1.091** | **0.105** |
| **abs2** | **1.078** | **0.179** | **0.308** | **0.911** | **1.037** | **1.092** | **0.104** |
|  |  |  |  |  |  |  |  |
| Date:24/08/2023 |  |  |  |  |  |  |  |
| type of cell:Hep-3B |  |  |  |  |  |  |  |
|  | **negative control** | **1000µg/ml** | **500µg/ml** | **300µg/ml** | **100µg/ml** | **50µg/ml** | **blank** |
| **abs1** | **1.351** | **0.315** | **1.26** | **1.288** | **1.34** | **1.353** | **0.105** |
| **abs2** | **1.354** | **0.328** | **1.286** | **1.271** | **1.334** | **1.399** | **0.104** |
|  |  |  |  |  |  |  |  |
|  |  |  |  |  |  |  |  |
| Date:24/08/2023 |  |  |  |  |  |  |  |
| type of cell:B16F1 |  |  |  |  |  |  |  |
|  | **negative control** | **1000µg/ml** | **500µg/ml** | **300µg/ml** | **100µg/ml** | **50µg/ml** | **blank** |
| **abs1** | **1.092** | **0.592** | **0.913** | **1.070** | **1.068** | **1.07** | **0.163** |
| **abs2** | **1.088** | **0.582** | **0.9** | **1.036** | **1.049** | **1.087** | **0.164** |
|  |  |  |  |  |  |  |  |
|  |  |  |  |  |  |  |  |
| Date:24/08/2023 |  |  |  |  |  |  |  |
| type of cell:LX-2 |  |  |  |  |  |  |  |
|  | **negative control** | **1000µg/ml** | **500µg/ml** | **300µg/ml** | **100µg/ml** | **50µg/ml** | **blank** |
| **abs1** | **1.277** | **0.979** | **1.050** | **1.146** | **1.249** | **1.292** | **0.163** |
| **abs2** | **1.264** | **987.000** | **1.098** | **1.151** | **1.289** | **1.299** | **0.164** |
